# Azurocidin-induced inhibition of oxygen metabolism in mitochondria is antagonized by heparin

**DOI:** 10.3892/etm.2014.1939

**Published:** 2014-08-29

**Authors:** LIANG WANG, ZHIYONG LIU, ZHE DONG, JIEYI PAN, XIAOCHUN MA

**Affiliations:** Department of Intensive Care Medicine, The First Affiliated Hospital of China Medical University, Shenyang, Liaoning 110001, P.R. China

**Keywords:** sepsis, heparin, azurocidin, mitochondrial oxygen metabolism function

## Abstract

Heparin is a potent blood anticoagulant that has been demonstrated to attenuate inflammatory responses in sepsis. Sepsis is considered to be a microcirculation-mitochondrial distress syndrome. Azurocidin (AZU), a protein with strong heparin-binding potential that induces inflammatory responses and apoptosis, has been shown to increase the permeability of endothelial cells and induce the prognosis of sepsis. However, the function of AZU in mitochondrial oxygen metabolism has yet to be reported. The aim of the present study was to investigate whether heparin exhibits an antagonistic effect on AZU-induced mitochondrial dysfunction in human umbilical vein endothelial cells (HUVECs) and to further investigate the possible underlying mechanisms. HUVECs were randomly assigned into blank control, AZU, heparin plus AZU and heparin groups. The blank control group were incubated with phosphate-buffered saline for 12 h, while the AZU group were incubated with 1 μg/ml AZU for 12 h. The heparin plus AZU group were incubated with 100 μg/ml heparin for 2 h, followed by the addition of 1 μg/ml AZU and incubation for 12 h. The heparin group were incubated with 100 μg/ml heparin for 12 h. Flow cytometry was used to determine the mitochondrial membrane potential, while electron microscopy was used to determine the mitochondrial morphology. Western blotting and quantitative polymerase chain reaction were used to determine the protein and mRNA expression levels of Cox II in the mitochondria, respectively. Western blotting was also used to evaluate the concentration of AZU in cytoplasm, along with immunofluorescence analysis. AZU was revealed to decrease the mitochondrial membrane potential, reduce cytochrome *c* oxidase subunit II expression and destroy the morphology of the mitochondria. Heparin exhibited an antagonistic function on these processes and inhibited the endocytosis of AZU by HUVECs. In conclusion, the results indicated that AZU inhibited the oxygen metabolic function in mitochondria, and this function was effectively antagonized by heparin via the inhibition of AZU endocytosis by HUVECs. Therefore, heparin may be a potential therapeutic agent for treating mitochondrial dysfunction in the future.

## Introduction

Heparin is a potent blood anticoagulant, and previous studies have demonstrated that it can attenuate inflammatory responses and organ function injury in sepsis (1–4). Sepsis is the systemic inflammatory response syndrome (SIRS) secondary to bacterial infection (5). SIRS is characterized by the activation of the inflammatory and coagulation systems, which can lead to generalized hypoperfusion, multiple organ failure and mortality. Previous studies have shown that septic plasma reduces the oxygen consumption of healthy mitochondria. In addition, sepsis severity has been shown to significantly correlate with mitochondrial function inhibition (6). Thus, sepsis is considered to be a microcirculation-mitochondrial distress syndrome (7).

Azurocidin (AZU), a protein with strong heparin-binding potential, is stored in neutrophil granules and is released following the stimulation of cells in proximity to endothelial cells. AZU is known to be a multifunctional inflammatory mediator with the ability to induce vascular leakage (8). In addition, AZU has been hypothesized to promote the release of inflammatory factors and inhibit apoptosis (9–11). A previous study demonstrated that plasma AZU levels were significantly higher in patients with severe sepsis or septic shock when compared with patients with a non-septic illness in the intensive care unit (12). Furthermore, AZU has been found to be associated with the severity of disease, and elevated AZU levels at admission were shown to correlate with an increased risk of mortality (13,14). Endothelial cells are able to uptake AZU, and the internalized protein is targeted to perinuclear compartments of the endothelial cells, where AZU colocalizes with the mitochondria (10).

Although increased AZU levels and the inhibition of mitochondrial function are associated with the severity of sepsis, the function of AZU in mitochondrial oxygen metabolism has yet to be reported. Therefore, the present study investigated mitochondrial oxygen metabolism function by analyzing the mitochondrial membrane potential (ΔΨ), mitochondrial morphology and the expression of cytochrome *c* oxidase subunit II (Cox II). In addition, the present study investigated the inhibitory effect of heparin on the endocytosis of AZU by human umbilical vein endothelial cells (HUVECs).

## Materials and methods

### Cell culture

HUVECs were obtained from the American Type Culture Collection (Manassas, VA, USA). The cells were cultured in Dulbecco’s modified Eagle’s medium (Invitrogen Life Technologies, Carlsbad, CA, USA) supplemented with 10% fetal calf serum (Invitrogen Life Technologies), 100 IU/ml penicillin (Sigma-Aldrich, St. Louis, MO, USA) and 100 μg/ml streptomycin (Sigma-Aldrich). The cells were grown on sterile tissue culture dishes and were passaged every three days using 0.25% trypsin (Invitrogen Life Technologies). The HUVECs were cultured to a density of 90% to prepare for the subsequent experiments.

### Experimental protocol

HUVECs were randomly divided into four groups. The blank control group were incubated with phosphate-buffered saline (PBS) in a 5% CO_2_ incubator at 37°C for 12 h. The AZU group were incubated with AZU (p20160; USCN Life Science, Inc., Wuhan, China), at a final concentration of 1 μg/ml, in a 5% CO_2_ incubator at 37°C for 12 h. The heparin plus AZU group were incubated with 100 μg/ml heparin (Qianhong Biochemistry Co., Ltd., Changzhou, China) in a 5% CO_2_ incubator at 37°C for 2 h, and were subsequently incubated with 1 μg/ml AZU in a 5% CO_2_ incubator at 37°C for 12 h. The heparin group were incubated with heparin, at a final concentration of 100 μg/ml, in a 5% CO_2_ incubator at 37°C for 12 h.

### JC-1 fluorescence analysis and flow cytometry

Following the cell treatment, HUVECs from each group were dyed with JC-1 to determine the mitochondrial membrane potential (ΔΨ). JC-1 is a membrane permeable lipophilic dye that manifests as J-aggregates in the mitochondrial matrix (red fluorescence) and as monomers in the cytoplasm (green fluorescence). During mitochondrial depolarization, the red J-aggregates form green monomers due to a change in the ΔΨ. Thus, the ΔΨ can be measured as the red fluorescence intensity ratio.

The JC-1 assay was performed as follows. HUVECs from each group were digested with 0.25% trypsin and collected by centrifugation at 1,000 × g for 5 min. After removing the supernatant, the HUVECs were washed twice with PBS and collected by centrifugation at 2,000 × g for 5 min. JC-1 working solution (500 μl; KeyGen BioTech, Nanjing, China) was added to the system and the cells were resuspended. Following incubation for 20 min at 37°C, the HUVECs were collected by centrifugation at 2,000 × g for 5 min and washed twice with 1X incubation buffer (KeyGen BioTech). The HUVECs were resuspended in 500 μl 1X incubation buffer and analyzed by flow cytometry (FACSCalibur™; BD Biosciences, Franklin Lakes, NJ, USA) to determine the ΔΨ.

### Isolation of mitochondria

HUVECs were collected by centrifugation at 800 × g for 3 min, and resuspended in ice-cold PBS. Following removal of the supernatant, the HUVECs were incubated with 1 ml mitochondrial separation reagent with phenylmethanesulfonyl fluoride (Beyotime Institute of Biotechnology, Haimen, China) on ice for 15 min. The HUVECs were homogenized 50 times and centrifuged at 600 × g at 4°C for 10 min. The supernatant was transferred to an additional centrifuge tube, which was centrifuged at 4°C at 11,000 × g for 10 min. Following removal of the supernatant, the precipitation comprised the isolated mitochondria.

### Electron microscopy

Isolated mitochondria were fixed in 4% glutaraldehyde at 4°C overnight, and immersed in 1% osmium tetroxide for 2 h. Following washing in PBS, the specimens were dehydrated as follows: 50% alcohol for 10 min; 70% alcohol for 10 min; 80% acetone for 10 min (twice); 90% acetone for 10 min (twice); and dry acetone for 10 min (twice). The specimens were embedded in SPI-Pon 812 (Structure Probe, Inc./SPI Supplies, West Chester, PA, USA), and 600–800-nm sections were prepared. The sections were dyed with uranyl acetate and lead citrate for 5 min, and the samples were viewed using a transmission electron microscope (JEM-1200EX; JEOL, Ltd., Akishima, Japan).

### Western blotting

Total protein for AZU detection was extracted from the cytoplasm of the HUVECs, while for Cox II expression determination, specimens were extracted from the mitochondrial matrix. Total proteins from the cells were extracted using cytoplasmic protein reagent A (Beyotime Institute of Biotechnology), while total proteins from the mitochondrial matrix were extracted using a mitochondrial lysate (Beyotime Institute of Biotechnology). The proteins were quantified using the Bradford method. Samples of 50 μg protein were separated by SDS-PAGE and transferred to polyvinylidene fluoride membranes (Millipore, Billerica, MA, USA). The membranes were incubated overnight at 4°C with antibodies against Cox II (ab79393; 1:100) and Cox IV (ab110272; 1:100; Abcam, Shanghai, China), AZU (sc-33129; 1:100) and β-actin (sc-130301; 1:500; Santa Cruz Biotechnology, Inc. Dallas, TX, USA). Following incubation with a peroxidase-coupled anti-mouse IgG (sc-2371; Santa Cruz Biotechnology, Inc.) at 37°C for 2 h, bound proteins were visualized using enhanced chemiluminescence (Pierce Biotechnology, Inc., Rockford, IL, USA) and detected using a BioImaging System (UVP, Inc., Upland, CA, USA). Relative protein expression levels were quantified using β-actin and Cox IV as the loading controls.

### Quantitative polymerase chain reaction (PCR) using the SYBR Green method

Quantitative PCR was performed using the SYBR Green PCR master mix (Applied Biosystems, Foster City, CA, USA) in a total volume of 20 μl on a Fast Real-Time PCR System (Exicycler 96; Bioneer Corporation, Daejeon, Korea). The conditions were as follows: 95°C for 30 sec, followed by 40 cycles at 95°C for 5 sec and 60°C for 30 sec. A dissociation step was performed to generate a melting curve to confirm the specificity of the amplification. Cox IV was used as the reference gene. The relative gene expression levels were represented as ΔCt = Ct gene − Ct reference, and the fold change of gene expression was calculated using the 2^−ΔΔCt^ method. Experiments were repeated in quadruplicate. The primer sequences were obtained from GenBank and were as follows: Cox II forward, 5′-TCCCCTTCTGCCTGACACCT-3′, and reverse, 5′-TTCCTACCACCAGCAACCCT-3′; Cox IV forward, 5′-GTTATCATGTGGCAGAAGCA-3′; and reverse, 5′-CCAGTAAATAGGCATGGAGTT-3′.

### Immunofluorescence analysis

HUVECs were fixed in 4% paraformaldehyde for 15 min and incubated with an antibody against AZU (1:50; sc-33129; Santa Cruz Biotechnology, Inc.) at 4°C overnight. The HUVECs were incubated with a fluorescein isothiocyanate-conjugated secondary antibody (F4512,Sigma-Aldrich) for 60 min at room temperature in the dark. The nuclei were dyed with DAPI (Cell Signaling Technology, Inc., Boston, MA, USA) for cellular localization, and the system was sealed by half a drop of anti-fluorescent quencher (Solarbio Science & Technology Co., Ltd., Beijing, China). The samples were viewed using a laser scanning confocal microscope (FV1000S-SIM/IX81; Olympus Corporation, Tokyo, Japan) at a magnification of ×600, from which the green fluorescence intensity was calculated. The cytoplasmic concentration of AZU was represented as the fluorescence intensity.

### Statistical analysis

SPSS version 19.0 for Windows (IBM, Armonk, NY, USA) was used for all statistical analyses. The Student’s t-test was used to compare the red fluorescence intensity ratio and the relative expression levels of the target proteins between any two groups. All the P-values were based on a two-sided statistical analysis and P<0.05 was considered to indicate a statistically significant difference.

## Results

### Mitochondrial membrane potential (ΔΨ) changes in each group

**Figure 1:**
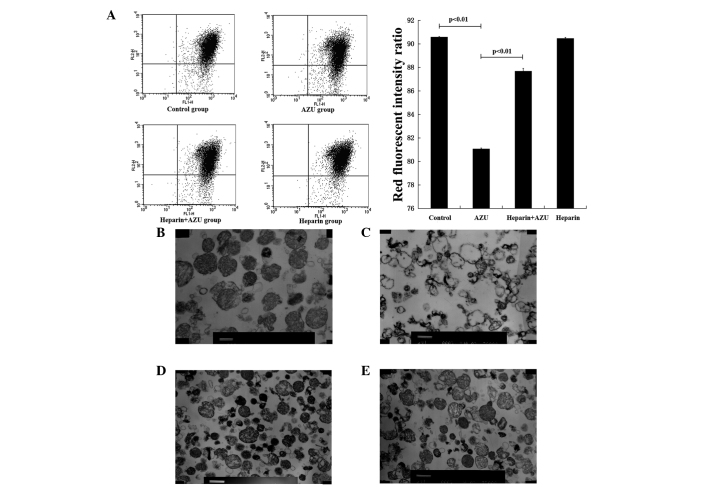
Mitochondrial membrane potential (ΔΨ) and morphology of the mitochondria. (A) Flow cytometry analysis was used to determine the ΔΨ. The horizontal axis (FL1-H) shows the green fluorescent intensity, while the vertical axis (FL2-H) shows the red fluorescent intensity. According to the door designation of FCM, four quadrants were established. Then the fact that the HUVECs were concentrated in UR (top and right) quadrant was revealed. The red fluorescence intensity ratio in the AZU group was much lower compared with the blank control group (P<0.01), while in the heparin plus AZU group, the ratio was higher compared with the AZU group (P<0.01). (B) Morphology of the mitochondria in the blank control group was almost normal. Electron microscopy showed that the matrix particles rarely disappeared and the crest was complete, with the inside and outside membrane structures legible. (C) The majority of the mitochondrial matrix density in the AZU group was severely reduced and exhibited vacuolation. The crest had parted or disappeared, and sections of the inside and outside membrane structures had vanished. (D) Mitochondrial matrix density in the heparin plus AZU group was reduced and the crest was complete. The integrity of the inside and outside membranes was reserved, and few mitochondria exhibited vacuolation. (E) The majority of the mitochondrial matrix density in the heparin group was almost normal, and the crest was complete with the inside and outside membrane structures legible. AZU, azurocidin.

Flow cytometry was employed to evaluate the ΔΨ in each group of HUVECs, and was measured as the red fluorescence intensity ratio. Compared with the blank control group (90.59±0.05, n=4), the ΔΨ was found to decrease in the AZU group (81.07±0.07; n=4; P<0.01). In the heparin plus AZU group (87.69±0.23, n=4), the ΔΨ was higher compared with that in the AZU group (81.07±0.07; n=4; P<0.01; Fig. 1A). These observations demonstrated that the ΔΨ was inhibited by AZU, and this function was antagonized by heparin.

### Changes to mitochondrial morphology in each group

Transmission electron microscopy was employed to evaluate the morphology of the mitochondria. The morphology of mitochondria in the blank control group was almost normal, with electron microscopy revealing that the matrix particles rarely disappeared and the crest was complete with the inside and outside membrane structures legible (Fig. 1B). The majority of the mitochondrial matrix density in the AZU group was severely reduced and exhibited vacuolation. The crest had parted or disappeared, and sections of the inside and outside membrane structures had vanished (Fig. 1C). The mitochondrial matrix density in the heparin plus AZU group was reduced and the crest was complete. In addition, the integrity of the inside and outside membranes was reserved, and few mitochondria showed vacuolation (Fig. 1D). The majority of the mitochondrial matrix density in the heparin group was almost normal. The crest was complete, with the inside and outside membrane structures legible (Fig. 1E). These observations demonstrated that AZU destroyed the structure of the mitochondria, but this function was antagonized by heparin.

### Expression levels of Cox II in the mitochondria

**Figure 2:**
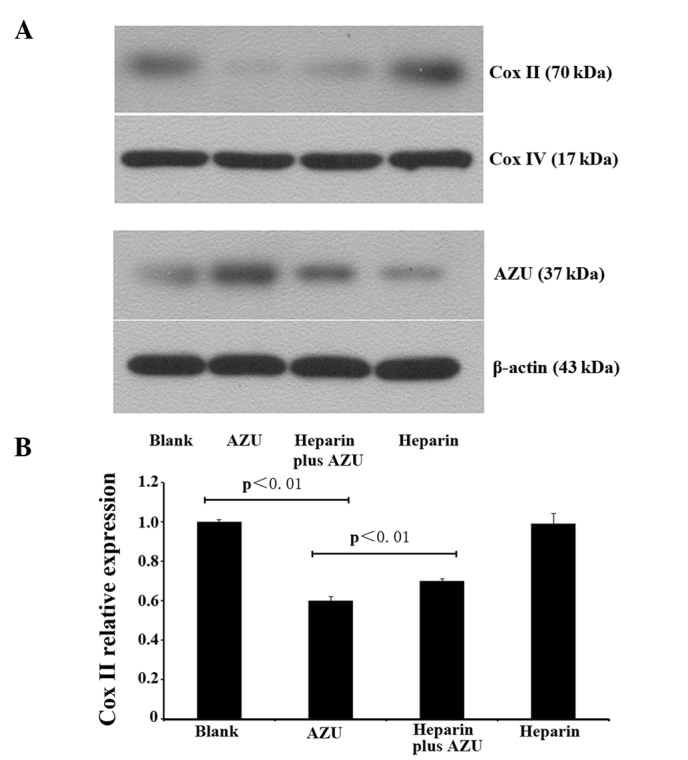
Western blotting and quantitative polymerase chain reaction (PCR) were used to detect the expression levels of Cox II and the endocytosis of AZU by human umbilical vein endothelial cell (HUVEC). (A) Western blotting showed that protein expression levels of Cox II in the AZU group were significantly decreased, while in the heparin plus AZU group, the levels were significantly increased compared with the AZU group. Due to the uptake of AZU by HUVECs, western blotting revealed that protein expression levels of AZU in the AZU group were significantly increased. By contrast, protein expression levels of AZU in the heparin plus AZU group were significantly decreased compared with the AZU group. (B) Quantitative PCR demonstrated that the mRNA expression levels of Cox II in the AZU group were significantly decreased (P<0.01), while in the heparin plus AZU group, the levels were significantly increased compared with the AZU group (P<0.01). AZU, azurocidin; Cox II, cytochrome *c* oxidase subunit II.

Protein expression levels of Cox II were analyzed by western blotting. Cox II expression levels were found to be lower in the AZU group compared with those in the blank control group. In addition, higher Cox II expression levels were observed in the heparin plus AZU group than in the AZU group (Fig. 2A). Quantitative PCR was employed to evaluate the mRNA expression levels of Cox II, and the results indicated that Cox II expression was lower in the AZU group (0.60±0.02; n=4) compared with the blank control group (1.00±0.01; n=4; P<0.01). Compared with the AZU group (0.60±0.02; n=4), Cox II expression in the heparin plus AZU group (0.70±0.01; n=4) was higher (P<0.01; Fig. 2B). These observations demonstrated that Cox II expression was inhibited by AZU, and this function was antagonized by heparin.

### Uptake and internalization of AZU by HUVECs

**Figure 3:**
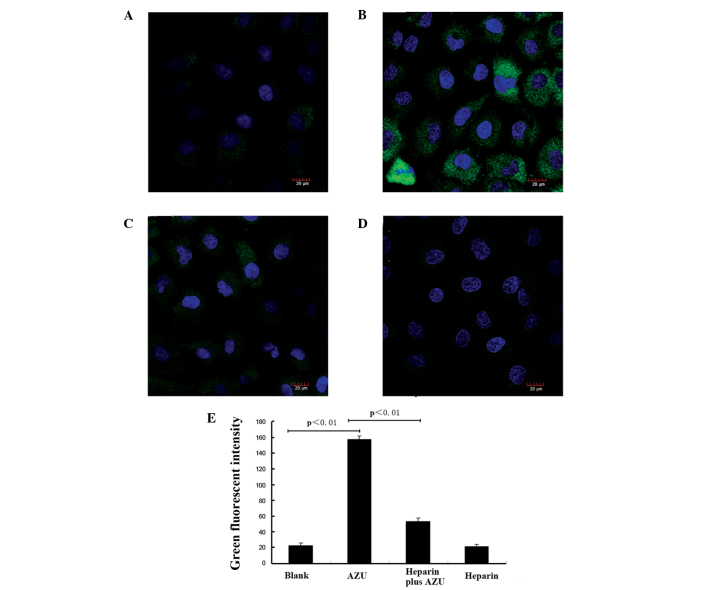
Immunofluorescence was used to detect the endocytosis of AZU by human umbilical vein endothelial cells (HUVECs) in each group. (A) Immunofluorescence analysis of the HUVECs revealed almost no green fluorescence in the cytoplasm of the blank control group. (B) By contrast, immunofluorescence analysis revealed that the highest intensity of green fluorescence was in the AZU group. (C) In the heparin plus AZU group, the intensity of green fluorescence was significantly reduced compared with the AZU group. (D) In the heparin group, almost no green fluorescence was detected in the cytoplasm of the HUVECs. (E) Quantitative fluorescence analysis detected the green fluorescence intensity in the cytoplasm, which was significantly higher in the AZU group compared with the blank control group (P<0.01). In the heparin plus AZU group, the cytoplasmic green fluorescence intensity was significantly decreased compared with the AZU group (P<0.01). AZU, azurocidin.

Immunofluorescence analysis was used to evaluate the concentration of AZU. The intensity of green fluorescence was deemed to be the concentration of AZU. HUVECs exhibited the lowest green fluorescence intensity in the cytoplasm of the blank control and heparin groups (Fig. 3A and D). By contrast, the AZU group has the highest intensity of green fluorescence (Fig. 3B). Compared with the AZU group, the intensity of green fluorescence was significantly reduced in the heparin plus AZU group (Fig. 3C). In order to evaluate the relative cytoplasmic concentration of AZU, quantitative fluorescence was used to detect the green fluorescence intensity in the cytoplasm. The cytoplasmic green fluorescence intensity in the AZU group was significantly higher (157.4±4.36; n=4) compared with the blank control group (22.3±3.12; n=4; P<0.01). In addition, the cytoplasmic green fluorescence intensity in the heparin plus AZU group (52.7±4.75; n=4) was significantly decreased compared with that in the AZU group (157.4±4.36; n=4; P<0.01; Fig. 3E).

Western blotting was also employed to evaluate the concentration of AZU. Since the expression of AZU in the cytoplasm is low, western blotting was used to determine the degree of AZU endocytosis. The protein expression levels of AZU in the AZU group were significantly increased, while the levels in the heparin plus AZU group were significantly decreased compared with those in the AZU group (Fig. 2A). These results demonstrated that the HUVECs were able to uptake and internalize AZU, with heparin exhibiting an antagonistic function in this process.

## Discussion

AZU is a multifunctional, inactive serine-protease homolog (15). Since the protein has a strong heparin binding potential, AZU is also known as heparin-binding protein. A previous study demonstrated that AZU has a high basic amino acid content and contains consensus sequences (-X-B-B-X-B-X and -X-B-B-B-X-X-B-X) that are known as glycosaminoglycan recognition sites (16). However, the mode of interaction between AZU and heparin remains unclear. The slightly different distribution of basic residues in AZU and the heterogeneity of heparin indicates that non-specific electrostatic forces form the main basis of the interactions (16). In the present study, HUVECs were shown to uptake AZU analogously. The expression of AZU is low in HUVECs, but the results revealed that the content was significantly increased following the addition of exogenous AZU, as demonstrated by western blotting and immunofluorescence analysis. In addition, heparin was shown to reduce the content of exogenous AZU in the cytoplasm.

Glycocalyx, considered to be an analog of heparin, is negatively charged with a mesh-like structure (17). AZU is hypothesized to bind to the glycoprotein, which allows for the uptake by endothelial cells. Subsequently, AZU causes the rearrangement of the endothelial cell cytoskeleton via the Rho-Rho-kinase pathway and increases the vascular permeability. In addition, AZU promotes the migration of neutrophils across the vascular wall, functioning as an inflammatory mediator (8,18,19). Glycocalyx plays a role as the binding site for AZU on the surface of endothelial cells (20). In the present study, heparin was considered to be an antagonist of glycocalyx by binding to AZU. AZU was shown to inhibit the function of mitochondrial oxygen metabolism, while heparin effectively reduced the uptake of AZU by endothelial cells. Heparin and glycocalyx were hypothesized to exhibit a competitive inhibition function against AZU binding to endothelial cells, since heparin and glycocalyx have a similar structure and charge characteristics.

Mitochondria are the respiratory and energetic centers of cells. Sepsis is considered as cytopathic hypoxia (21), and different experimental models support an underlying role of cytopathic hypoxia in mitochondrial dysfunction. Mitochondrial complex IV (cytochrome *c* oxidase) is responsible for the final oxygen reduction into water. The mitochondrial membrane potential (ΔΨ) is also considered as the function of mitochondrial oxygen metabolism. The results of the present study indicated that AZU reduced the expression of Cox II, which is a subunit of Cox that is mitochondrial encoded only. AZU was also shown to reduce the ΔΨ and destroy the morphology of the mitochondria. Thus, AZU was hypothesized to destroy mitochondrial function and morphology, and inhibit the oxygen metabolic function of mitochondria by reducing the synthesis of key enzymes involved in mitochondrial oxygen metabolism.

The import of proteins into the mitochondria is controlled by several membrane-associated translocation complexes, including the translocase of the outer membrane (TOM)complex, the translocase of the inner membrane (TIM) complex, the sorting and assembly machinery complex for the insertion of β-barrel proteins into the outer membrane and the presequence translocase-associated motor complex that drives preprotein translocation into the matrix. These mitochondrial protein translocases, as well as specific cytosolic chaperones, recognize targeting information in proteins requiring importation, such as the N-terminal presequence and molecular charge, and assist with unfolding, membrane insertion or translocation and refolding/assembly in the mitochondria (22). In the present study, AZU was shown to inhibit the expression of Cox II, which exists only in the matrix of mitochondria. Cox II has a positive molecular charge and a β-barrel structure; thus, AZU has the structure foundation for translocation into the mitochondria via the TOM/TIM pathway, after which AZU was shown to affect protein function in the mitochondrial matrix. However, further experiments are required to confirm this hypothesis.

In conclusion, the present study has demonstrated that AZU inhibits the oxygen metabolic function in mitochondria. This function was effectively antagonized by heparin via the inhibition of AZU endocytosis by HUVECs. Therefore, heparin may be a potential therapeutic agent for treating sepsis-induced mitochondrial dysfunction in the future.
